# Evaluation of *Bacillus* spp. as Potent Probiotics with Reduction in AHPND-Related Mortality and Facilitating Growth Performance of Pacific White Shrimp (*Litopenaeus vannamei*) Farms

**DOI:** 10.3390/microorganisms11092176

**Published:** 2023-08-29

**Authors:** Porranee Proespraiwong, Rapeepat Mavichak, Kentaro Imaizumi, Ikuo Hirono, Sasimanas Unajak

**Affiliations:** 1 Department of Biochemistry, Faculty of Science, Kasetsart University, Bangkok 10900, Thailand; porranee.pro@cpf.co.th (P.P.); kentaro.i@ku.th (K.I.); 2 Kasetsart Vaccines and Bio-Product Innovation Centre, Kasetsart University, Bangkok 10900, Thailand; 3 Charoen Pokphand Foods Public Co., Ltd., Aquatic Animal Health Research Center, Samut Sakhon 74000, Thailand; rapeepat.mav@cpf.co.th; 4 Graduate School of Marine Science and Technology, Tokyo University of Marine Science and Technology, Konan 4-5-7, Minato-Ku, Tokyo 108-8477, Japan; hirono@kaiyodai.ac.jp

**Keywords:** shrimp, probiotics, aquaculture, acute hepatopancreatic necrosis disease (AHPND), *Bacillus* spp., nursery trial, antimicrobial peptide

## Abstract

Acute hepatopancreatic necrosis disease (AHPND) is a serious bacterial disease affecting shrimp aquaculture worldwide. In this study, natural microbes were used in disease prevention and control. Probiotics derived from *Bacillus* spp. were isolated from the stomachs of AHPND-surviving Pacific white shrimp *Litopenaeus vannamei* (22 isolates) and mangrove forest soil near the shrimp farms (10 isolates). *Bacillus* spp. were genetically identified and characterized based on the availability of antimicrobial peptide (AMP)-related genes. The phenotypic characterization of all *Bacillus* spp. was determined based on their capability to inhibit AHPND-causing strains of *Vibrio parahaemolyticus* (VP_AHPND_). The results showed that *Bacillus* spp. without AMP-related genes were incapable of inhibiting VP_AHPND_ in vitro, while other *Bacillus* spp. harboring at least two AMP-related genes exhibited diverse inhibition activities. Interestingly, K3 [*B. subtilis* (*srfAA^+^* and *bacA^+^*)], isolated from shrimp, exerted remarkable inhibition against VP_AHPND_ (80% survival) in Pacific white shrimp and maintained a reduction in shrimp mortality within different ranges of salinity (75–95% survival). Moreover, with different strains of VP_AHPND_, *B. subtilis* (K3) showed outstanding protection, and the survival rate of shrimp remained stable among the tested groups (80–95% survival). Thus, *B. subtilis* (K3) was further used to determine its efficiency in shrimp farms in different locations of Vietnam. Lower disease occurrences (2 ponds out of 30 ponds) and greater production efficiency were noticeable in the *B. subtilis* (K3)-treated farms. Taking the results of this study together, the heat-shock isolation and genotypic-phenotypic characterization of *Bacillus* spp. enable the selection of probiotics that control AHPND in Pacific white shrimp. Consequently, greater disease prevention and growth performance were affirmed to be beneficial in the use of these probiotics in shrimp cultivation, which will sustain shrimp aquaculture and be environmentally friendly.

## 1. Introduction

Acute hepatopancreatic necrosis disease (AHPND) is one of the most serious threats to shrimp farming [1]. AHPND is caused mainly by *Vibrio parahaemolyticus* (VP_AHPND_), which carries plasmid encoding-specific toxin genes [2]. This pathogen affects penaeid shrimp, including the Pacific white shrimp *Litopenaeus vannamei* and black tiger shrimp *Penaeus monodon*, with mortality up to 100% within 20 to 30 days of cultivation, resulting in significant economic losses in shrimp aquaculture [1,3]. Although the use of antibiotics is effective against this bacterial disease, the risk of resistance against drugs among bacteria in the environment requires alternative strategies to address this disease.

In general, bacteria, which are promising contributors to shrimp health, are widely applied in shrimp farms for health management as an alternative strategy to reduce the risk of diseases [4]. Probiotics, ‘live microorganisms which when administered in adequate amounts confer a health benefit on the host’ [5], and biological control agents [6] have been studied in the research field of aquaculture. Previous studies suggest that oral administration of exogenous bacteria stimulates shrimp immune reactions [7,8], and bacterial antagonism occurs in the environment, including the shrimp gut and pond water [9,10]. However, the exact mechanism of the probiotic effects of bacteria on shrimp and the appropriate application are uncertain.

*Bacillus* spp. is one of the most studied and used bacteria as a probiotic or biocontrol agent in aquaculture [11]. In shrimp, oral administration of spores or vegetative cells of specific strains of *Bacillus* spp. reduces the mortality of shrimp caused by bacterial infections, with induction of the host immune system and/or antagonism between the bacteria as possible mechanisms [7,8,12,13,14,15]. Antimicrobial peptides (AMPs) secreted by *Bacillus* spp., such as bacillomycin, fengycin, iturin, surfactin, bacilysin, and subtilin, inhibit the growth of other microorganisms, especially bacteria and fungi [16,17]. The risk of resistance among bacteria against those AMPs is thought to be small [18]; thus, the application of AMP-producing *Bacillus* to aquaculture fields is promising.

The stomach microbiota of penaeid shrimp may play a crucial role in protecting against bacterial infections. *Vibrio* bacteria, such as VP_AHPND_ and *Vibrio penaeicida*, colonize the shrimp’s stomach during the initial stages of infection [19,20]. Moreover, in contrast to mammalian animals, shrimp stomachs host a diverse range of bacteria [21,22], with variations observed in the presence or absence of AHPND development [1,23]. This knowledge suggests that the bacterial community in the stomach microbiota of shrimp, similar to the intestinal microbiota of mammals, might confer beneficial effects on shrimp.

Southeast Asian countries have been heavily impacted by AHPND [24]. However, interestingly, not all shrimp farms or shrimp ponds were affected by the disease. Thus, we expected that shrimp may have specific factors that reduce the risk of AHPND. In this study, *Bacillus* spp. were isolated from the shrimp stomach and environment, and their potential as beneficial bacteria for shrimp was evaluated.

## 2. Materials and Methods

### 2.1. Bacillus *spp.* Isolation

*Bacillus* spp. in this study were isolated from two sources: (1) from the stomachs of surviving shrimp from ponds that were positive for AHPND outbreaks on local farms in Samut Songkhram Province and (2) from soils in mangrove forests in Thailand. The isolation of *Bacillus* spp. followed the heat-cold shock method [25]. Briefly, stomachs of AHPND-surviving shrimp and soils from mangroves were ground and diluted in 0.85% Normal saline solution (NSS) and then heated at 80 °C for 20 min before rapidly chilling on ice for 1 to 2 min. Suspensions were serially diluted in 0.85% NSS and spread on tryptic soy agar (TSA). After incubation at 30 °C for 20 h, *Bacillus*-like colonies were selected on the basis of their morphology and kept at −80 °C until use.

### 2.2. VP_AHPND_ Isolation and Identification

To obtain the *V*. *parahaemolyticus* (VP) AHPND strain (VP_AHPND_), the stomachs of diseased shrimp from different areas in the eastern and southern areas of Thailand were collected, including Chanthaburi (CT), Rayong (RY), Trat (TR), Nakhon Si Thammarat (NK), Surat Thani (SR), Chumphon (CP), and Songkhla (SK). The shrimp stomachs were aseptically dissected, minced, and serially diluted 10-fold in 0.85% NaCl (normal saline; NSS), spread on thiosulfate citrate bile salt (TCBS) agar, and incubated at 30 °C overnight [26]. Individual green colonies were chosen and identified using specific primers for VP [27] and VP_AHPND_ [28]. One VP isolate from each province that was positive with both primer sets was examined for pathogenicity in a shrimp challenge assay. VP isolates that caused AHPND were designated VP_AHPND_ hereafter. VP_AHPND_ isolates were collected in glycerol stocks and stored at −80 °C.

### 2.3. Phenotypic Characterization of Bacillus *spp.*

#### 2.3.1. In Vitro Inhibition Assay: Solid Medium

The soft agar overlay technique was used to determine inhibition on a solid medium [29]. Soft agar was prepared by mixing melted TSA and TSB at a ratio of 1:2. Then, 3 mL of soft agar was mixed with 50 µL of overnight cultured VP_AHPND_. The mixture was poured onto the surface of solidified TSA in 87 mm diameter Petri dishes and left for 20 to 30 min to solidify. Then, blank antimicrobial susceptibility disks (Oxoid) were placed on the surface of the agar, and 10 µL of 1 × 10^8^ CFU/mL of each *Bacillus* culture was dropped on the blank disks. After incubation at 30 °C for 16 h, the diameters of the clear zones surrounding the disks were measured.

#### 2.3.2. In Vitro Inhibition Assay: Liquid Medium

Overnight cultures of different *Bacillus* spp. were diluted to 1 × 10^5^ CFU·mL^−1^ in 10 mL of TSB. The overnight culture of VP_AHPND_ was prepared as a 100-fold dilution with *Bacillus* culture and shaken at 200 rpm 30 °C for 20 h. TSB was used as the control (without *Bacillus*). For the negative control, TSB was not inoculated with either *Bacillus* or VP_AHPND_. Samples with different *Bacillus* isolates were 10-fold serially diluted in 0.85% NSS and spread on TCBS to count VP_AHPND_ and TSB to count *Bacillus* spp. All assays were performed in triplicate for each *Bacillus* isolate.

#### 2.3.3. Antibiotic Susceptibility of *Bacillus* spp.

The soft agar overlay technique was used to examine antibiotic susceptibility inhibition on a solid medium [29]. The preparation of soft agar followed the method described above. A mixture of soft agar containing *Bacillus* isolates was poured on the surface of solidified TSA and allowed to solidify. Once solidified, 11 antibiotic discs (Oxoid), including amoxicillin (10 µg), oxytetracycline (30 µg), sulfa-trimethoprim (25 µg), doxycycline (30 µg), erythromycin (15 µg), gentamycin (10 µg), enrofloxacin (5 µg), tetracycline (30 µg), ceftriaxone (30 µg), streptomycin (10 µg) and norfloxacin (10 µg), were placed on the surface of solid agar. After incubation at 30 °C for 16 h, the diameter of the clear zone surrounding the antibiotic discs was measured and interpreted as susceptible (S), intermediate (I), and resistant (R) following the standards of the Clinical and Laboratory Standards Institute [30].

### 2.4. Genotypic Characterization of Bacillus *spp.*

#### 2.4.1. Species Identification

The species identification of isolated *Bacillus* spp. was based on the entire sequence of 16S rRNA gene (1500 bp) using universal primer 8f and 1490r as described below. The isolated *Bacillus*-like colonies were cultured in tryptic soy broth (TSB) at 30 °C for 20 h. Bacterial DNA was extracted for species identification following the standard phenol–chloroform extraction method. Primers 8f (3′GAGTTTGATCCTGTGCTCAG5′) and 1490r (5′GACTTACCAGGGTATCTAATCC-3′) were used as 16S rRNA universal primers for the bacteria. PCR products were purified with a GeneJET PCR Purification Kit (Thermo Fisher Scientific, Waltham, MA, USA) and sequenced using Sanger sequencing using a 3730XL DNA Analyzer (Applied Biosystems, Waltham, MA, USA). Bacterial identification was performed using NCBI BLAST (https://blast.ncbi.nlm.nih.gov/Blast.cgi, 20 April 2020).

#### 2.4.2. Antimicrobial Peptide (AMP)-Related Gene Determination

Genomic DNA of *Bacillus* spp. was used as a template for examining the presence of AMP genes, including *bmyB* (bacillomycin L synthetase B), *fenD* (fengycin synthetase), *ituC* (iturin A synthetase C), *srfAA* (surfactin synthetase subunit 1), *bacA* (bacilysin biosynthesis protein), and *spaS* (subtilin) [31](Mora et al., 2011). All PCR amplifications were performed in 25 μL reactions containing 100 μg of genomic DNA template, 2.5 μL of 10x DreamTaq Buffer, 0.2 mM dNTP (Thermo Scientific, Hong Kong, China), 0.5 μM of each primer (Table 1), and 0.6 U of DreamTaq DNA Polymerase (Thermo Scientific, Hong Kong, China). All reactions were run on a MyCycler (Bio-Rad, Hong Kong, China). The cycling conditions for the amplification of all targets were as follows: initial denaturation at 95 °C for 2 min; 35 cycles of 95 °C for 30 s, 55 °C for 30 s and 72 °C for 40 s; and a final extension at 72 °C for 5 min. The PCR amplicons were analyzed in a 2% (*w*/*v*) agarose gel.

### 2.5. In Vivo AHPND Challenge Test and Efficacy Analysis

#### 2.5.1. Experimental Shrimp

Healthy Pacific white shrimp were kindly provided by Aquatic Animal Research Mae Klong, Charoen Pokphand Foods Public Co., Ltd. (CP), Bangkok, Thailand. Shrimp were maintained in aerated aquaculture tanks at Kasetsart University until the challenge test. The shrimp were randomly screened for the presence of AHPND, EHP, WSSV, IHHNV, TSV, and YHV using PCR based on a previous method [28,32,33,34,35,36]. Shrimp conditions were maintained during the experiment as follows: pH 7.8–8.2, temperature 28–32 °C, salinity 20 ppt, alkalinity 170–190 mg, TAN less than 1 ppm, and NO_2_^−^ less than 1 ppm.

#### 2.5.2. Preparation of *Bacillus* spp. and VP_AHPND_

*Bacillus* isolates and VP_AHPND_ in glycerol stocks were streaked on TSA and TCBS agar, respectively. After incubation at 30 °C overnight, a single colony was inoculated in TSB and shaken at 250 rpm and 30 °C for 20 h. The bacterial amount was quantified using the plate count method on TSA and TCBS agar.

#### 2.5.3. Pathogenicity Analysis of Isolated VP_AHPND_ in Shrimp

Three hundred sixty shrimp, 0.5 ± 0.03 g, were acclimated in aerated experimental 200-L tanks for 3 days. The shrimp were separated into two groups: VP_AHPND_ challenge and control group, 60 shrimp in each group with triplicate. In the challenge groups, shrimp were inoculated with isolated VP_AHPND_ at 10^4^ CFU·mL^−1^ using the immersion method [37]. In the control group, shrimp were inoculated with 100 mL of TSB. The hepatopancreas of moribund shrimps were examined for the presence of VP_AHPND_ using PCR [28], and histomorphology was determined using H&E staining. The number of dead shrimp was observed every 24 h after the challenge until 10 days after the challenge. All assays were performed in triplicate for each VP_AHPND_ isolate.

#### 2.5.4. Efficiency of Isolated Bacillus in Controlling AHPND: Laboratory Level

Three hundred sixty shrimp, 0.5 g ± 0.03 g, were kept in a 400-L container with aeration, and the diseased contaminant was determined prior to testing. To test the disease control, 60 shrimps in each group were directly immersed in different *Bacillus* isolates (including K3, K5, K6, K11, K12, K13, K19, P4, and P6) at 1 × 10^5^ CFU·mL^−1^ for 10 h. After that, VP_AHPND_ at 1 × 10^4^ CFU·mL^−1^ was immediately added to the aquarium. For the control group, 60 shrimp were treated with TSB instead of *Bacillus* spp. All experiments were performed in triplication. Moribund shrimp were examined for the presence of VP_AHPND_ using PCR. The number of dead shrimp was observed every 24 h. All assays were performed in triplicate for each *Bacillus* isolate.

#### 2.5.5. Evaluation of AHPND Control Efficiency at Different Salinities

Shrimp were kept in tanks with salinities of 5, 20, and 40 ppt. Shrimp were immersed in *Bacillus* spp. at 1 × 10^5^ CFU·mL^−1^ for 10 h and then challenged with VP_AHPND_ at 1 × 10^4^ CFU·mL^−1^ using the immersion method. The other methodology of this experiment was the same as that mentioned above. All assays were performed in triplicate for each salinity.

#### 2.5.6. Validation of Disease Control Efficiency against Different Strains of VP_AHPND_

The efficacy of *Bacillus* sp. against various strains of VP_AHPND_ was evaluated. Shrimp were immersed in 1 × 10^5^ CFU·mL^−1^ *Bacillus* sp. for 10 h and then challenged with 1 × 10^4^ CFU·mL^−1^ of different VP_AHPND_ strains (strains CT, RY, TR NK, SR, CP, and SK) in a salinity of 20 ppt. The other methodology of this experiment was the same as that mentioned above. All assays were performed in triplicate for each VP_AHPND_ isolate.

#### 2.5.7. Efficiency of Isolated Bacillus in Controlling AHPND: Field Level

Field trials were conducted in nursery farms in Quang Binh, Binh Dinh, Ninh Thuan, Ben Tre, Bac Lieu, and Kien Giang at local farms in Vietnam (Supplementary date S1). Healthy postlarvae were reared in 250 m^3^ aerated aquaculture tanks at a density of 1600 (pcs/m^3^) for 35 days. Shrimp were initially randomly screened for the presence of AHPND, EHP, WSSV, IHHNV, TSV, and YHV using PCR based on previously described methods. Culture conditions for shrimp were maintained during the whole experiment as follows: pH at 7.8–8.2, temperature at 28–32 °C, salinity at 20 ppt, alkalinity at 170–190 mg, TAN less than 1 ppm and NO_2_^−^ less than 1 ppm. In the treatment group (30 tanks), *Bacillus* spp. was added directly to the water at a dose of 10^5^ CFU·mL^−1^ every 3 days. In the control group (30 tanks), postlarvae did not receive any probiotics. The number of final shrimp (pcs), total weight (kg), final size (pcs·kg^−1^), total feed (kg), FCR, % survival, and VP_AHPND_ infection were recorded at the end of the experiment.

#### 2.5.8. Statistical Analysis

In bacterial inhibition analysis, Dunnett’s multiple comparisons test was performed to compare the bacterial number in the test groups and the control. In challenge tests, the survival of shrimp was analyzed using the log-rank test. These statistical analyses and figure preparations were conducted using GraphPad Prism v.6 (GraphPad).

## 3. Results

### 3.1. Bacillus *spp.* Isolation

To collect the *Bacillus* spp., the stomachs of AHPND-surviving shrimp and the soil from the AHPND outbreak area were targeted for *Bacillus* spp. isolation. After heat and cold shock treatment, *Bacillus* spp. were entered into the sporulation stage, while other bacteria were killed. Thus, the spore suspension was spread on solid agar to allow the *Bacillus* spp. to re-enter the vegetative stage. The bacteria showing different morphological characteristics of the colonies were collected, and their species were determined based on 16S rRNA analysis. A total of 22 isolates, designated K1–K22, were isolated from AHPND-surviving shrimp stomachs, whereas 10 isolates, named P1–P10, were isolated from the soil in mangrove forests (Table 1).

### 3.2. VP_AHPND_ Isolation, Identification, and Pathogenicity of Isolated VP_AHPND_

VP_AHPND_ was isolated from the stomachs of naturally AHPND-infected shrimp in commercial shrimp farms. Seven strains were collected from different provinces in Thailand: Chanthaburi (CT), Rayong (RY), Trat (TR), Nakhon Si Thammarat (NK), Surat Thani (SR), Chumphon (CP), and Songkhla (SK). Those strains were confirmed using PCR using specific primers for *V*. *parahaemolyticus* and VP_AHPND_. The pathogenicity of these isolates demonstrated acute mortality, with survival rates ranging from 40.56–67.78% (Supplementary date S2). The mortality of AHPND-challenged shrimp ceased at 6–8 days after infection. The presence of VP_AHPND_ in the hepatopancreas of moribund shrimp was confirmed using PCR, and the AHPND histomorphology was confirmed using H&E staining (Supplement data S1 and S2). Pathogenic bacteria were reisolated from dead shrimp, demonstrating authentic infection using the tested VP_AHPND._

### 3.3. Phenotypic Characterization of Bacillus *spp.*

#### 3.3.1. In Vitro Inhibition Assay: Solid Medium and Liquid Medium

**Figure 1:**
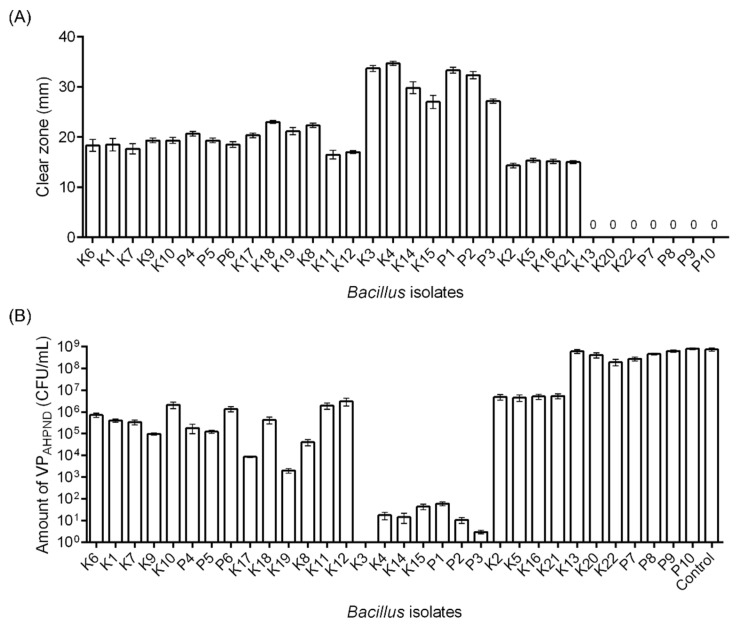
Growth inhibition analysis of *Bacillus* spp. against VP_AHPND_. (**A**) Solid agar; isolated *Bacillus* spp. were grown on the lawn of VP_AHPND_ (strain RY) on solid agar plates. The inhibition zone was measured and demonstrated as the diameter of the clear zone (mm). (**B**) Liquid medium; *Bacillus* spp. were cocultured overnight with VP_AHPND_ (strain RY). The control is the culture without *Bacillus*. The order corresponds to Table 2, which shows the presence of AMP-related genes.

To assess the inhibition activity of *Bacillus* isolates against VP_AHPND_ in vitro, the width of the inhibition zone on agar plates and the growth of bacteria in liquid media were evaluated. The twenty-five *Bacillus* isolates exhibited inhibition zones ranging from 14.33–34.67 mm against VP_AHPND_ (strain RY, topic 3.4). Isolates K3, K4, K14, K15, P1, P2, and P3 displayed outstanding inhibition with clear zone diameters ranging from 27.00–34.67 mm (Figure 1A).

In liquid media, *Bacillus* isolates showed significant (Dunnett’s multiple comparisons test, *p* < 0.001) inhibition against the growth of VP_AHPND_, except isolates K13, P9, and P10 (Figure 1B). These results concurred with the inhibition assay on a solid medium.

#### 3.3.2. Antibiotic Susceptibility of *B. subtilis* (K3)

*B. subtilis* (K3) was susceptible to almost all the tested antibiotics except for amoxicillin (intermediate), oxytetracycline (intermediate), and streptomycin (intermediate) (Table 3).

### 3.4. Genotypic Characterization of Bacillus *spp.*

#### 3.4.1. Species Identification

Upon 16S rRNA identification, the nucleotide sequences (1500 bp) from the isolated *Bacillus* spp. were compared with references sequenced in the NCBI database to identify their species. In conclusion, the populations of isolated *Bacillus* species were *B. subtilis* (11 isolates), *B. amyloliquefaciens* (5 isolates), *B. velezensis* (4 isolates), and *B. licheniformis* (4 isolates) (Table 3).

#### 3.4.2. Antimicrobial Peptide (AMP)-Related Gene Determination

To determine the inhibition activity of isolated *Bacillus* spp. against VP_AHPND_, six different AMP genes were tested. Analysis based on gene-specific PCR detection showed diverse distributions of AMP genes in different *Bacillus* isolates (Table 2). From a total of 32 isolates, the *srfAA* gene was most frequently found (25 isolates), followed by *bacA* (19 isolates), *bmyB* (11 isolates), *fenD* (11 isolates), and *ituC* and *spaS* (1 isolate). More than half of the isolates (21 isolates) harbored at least two of the tested AMP genes, while seven isolates had none of the tested genes. The most frequent patterns of AMP genes were *srfAA*^+^-*bacA*^+^ (7 isolates) and *srfAA*^+^-*bacA*^+^-*bmyB*^+^-*fenD*^+^ (7 isolates). *Bacillus* isolates carrying AMP genes contained at least *srfAA*^+^.

### 3.5. In Vivo Efficacy Analysis of Bacillus against AHPND

#### 3.5.1. Efficiency of Isolated Bacillus in Controlling AHPND: Laboratory Level

**Figure 2:**
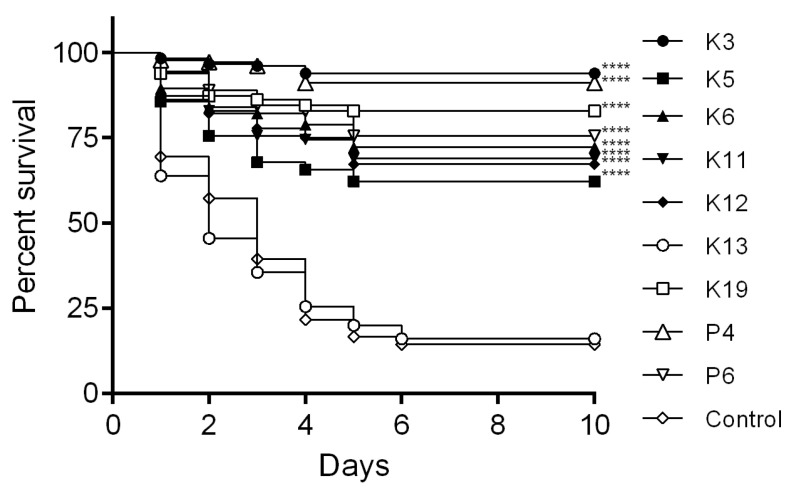
Evaluation of AHPND disease control by *Bacillus* spp. Shrimp were treated with different *Bacillus* spp. isolates (isolates K3, K5, K6, K11, K12, K13, K19, P4, and P6) for 10 h following challenge by immersion with 10^4^ CFU·mL^−1^ VP_AHPND_ (strain RY). **** *p* < 0.0001 compared with the control.

Challenge tests were performed to evaluate the capability of *Bacillus* isolates P4, P6, K3, K5, K6, K11, K12, K13, and K19 to reduce shrimp mortality during an artificial challenge by VP_AHPND_ (strain RY). Shrimp were treated with different *Bacillus* isolates (isolates P4, P6, K3, K5, K6, K11, K12, and K19) and showed significantly higher survival rates than the control (*p* < 0.0001). However, K3 (*B. subtilis*) exerted the highest effectiveness in reducing shrimp mortality. Isolate K13 (*B. subtilis*), which lacks the tested AMP genes, did not show a significant difference compared to the control (Figure 2).

#### 3.5.2. Evaluation of AHPND Control Efficiency at Different Salinities

**Figure 3:**
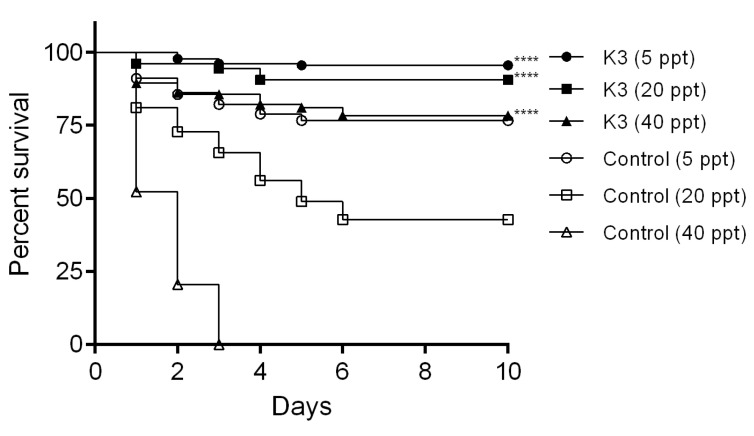
Effect of water salinity on *B. subtilis* (K3) efficiency in controlling AHPND. Shrimp were treated with *B. subtilis* (K3) for 10 h following the challenge with VP_AHPND_ (strain RY) at 10^4^ CFU·mL^−1^ by the immersion method. Shrimp were reared at different salinities, including 5 ppt, 20 ppt, and 40 ppt. **** *p* < 0.0001 compared with the control for each salinity.

To ensure that *B. subtilis* (K3) had the highest AHPND control effectiveness, whether it remained effective at different salinities was evaluated. The challenge test was monitored after treatment with *B. subtilis* (K3). Survival rates of shrimp 76.67% (5 ppt), 42.78% (20 ppt), and 0% (40 ppt) were observed in control shrimp after immersion with VP_AHPND_ (strain RY) without pretreatment with *B. subtilis* (K3) (Figure 3). This result indicated that the virulence of VP_AHPND_ (strain RY) was salinity-dependent. However, after treatment with *B. subtilis* (K3), a significantly higher survival rate than the control groups for each salinity (*p* < 0.0001) was observed (Figure 3).

#### 3.5.3. Validation of Disease Control Efficiency against Different Strains of VP_AHPND_

**Figure 4:**
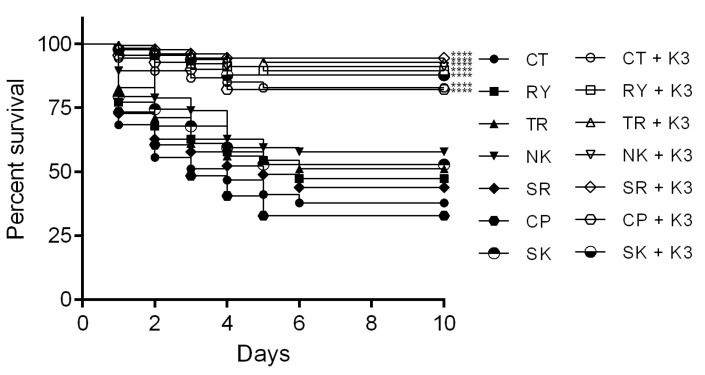
Efficiency of *B. subtilis* (K3) in controlling different strains of VP_AHPND_. Shrimp treated with *B. subtilis* (K3) for 10 h following challenge with different strains of VP_AHPND_ (strains CP, CT, SK, RY, NK, TR, and SR) at 10^4^ CFU·mL^−1^. **** *p* < 0.0001 compared with the control without *Bacillus*.

To determine the efficiency of *B. subtilis* (K3) in protection from different VP_AHPND_ strains, the survival rates of shrimp challenged with different VP_AHPND_ strains were observed in shrimp treated with 10^5^ CFU·mL^−1^ *B. subtilis* (K3) at 20 ppt salinity. Similar survival rates among different strains of VP_AHPND_ were observed as follows: CP (82.22%), CT (82.78%), SK (87.78%), RY (89.44%), NK (91.11%), TR (92.78%) and SR (94.44%); all tested groups exhibited significantly greater survival rates than the controls (32.78%, 37.78%, 52.78%, 47.22%, 57.78%, 51.11% and 43.89%, respectively) (Figure 4). The PCR determination of VP_AHPND_ and histomorphology of diseased shrimp confirmed the pathogenicity caused by AHPND disease (Supplementary Figures S3 and S4).

#### 3.5.4. Efficiency of Isolated Bacillus in Controlling AHPND: Field Level

Field trials were performed in a local nursery farm in Vietnam to evaluate the protection efficacy of *B. subtilis* (K3). Shrimp ponds that were treated with *B. subtilis* (K3) showed a low number of AHPND occurrences, and if eventually positive for AHPND, those ponds can be continually cultivated until harvested. Moreover, with *B. subtilis* (K3) threat, higher weight with lower FCR compared to the control was observed, which led to greater shrimp production (Table 4).

## 4. Discussion

Probiotics in aquaculture are a well-known method to improve the health status of aquatic animals; thus, the use of probiotics to control disease has been widely discussed. However, the validity of their use should be confirmed using rational probiotic screening and selection, as well as the elucidation of their efficiency in laboratory and field trials.

Upon AHPND devastation, we noticed that some shrimp survived in the AHPND-positive ponds. Hence, the ecosystem of the microbiome in the shrimp digestive system might reflect the regulation of the population of pathogenic bacteria by healthy bacteria. Therefore, probiotics, *Bacillus* spp., were isolated from two different sources: (1) from the surviving shrimp from the AHPND outbreak pond and (2) from the soils in mangrove forests in Thailand.

By using the heat-cold shock method, *Bacillus* spp. can be isolated from other bacterial species [25]. Based on genotypic and phenotypic identification, AMP identification was used to screen and group all *Bacillus* spp. AMP synthesis-related genes tested in this study have long been known to be responsible for the inhibitory activity of *Bacillus* spp. against other microorganisms [31], and it is not surprising that various *Bacillus* isolates possess these genes. However, a diverse pattern of AMPs containing *Bacillus* spp. was found, and it is not appropriate to classify them regarding the presentation of AMPs. In addition, the phenotypic determination of their capability to inhibit and control VP_AHPND_ growth and pathogenesis in vitro and in vivo facilitated the selection of candidate *Bacillus* spp. for further analysis.

*B. subtilis* (K3) markedly reduced shrimp mortality both at the laboratory level and at the farm level. The bacteria harbor *srfAA* and *bacA* genes. The *BacA* gene is known to contribute to the synthesis of bacilysin, but insufficient reports on controlling or inhibiting Gram-negative bacteria, including *Vibrio* spp., have been noted. Conversely, many inhibitory effects of *srfAA*-mediated surfactin against *Vibrio* were reported [38,39,40]. Therefore, it is expected that surfactin secreted by *B. subtilis* (K3) would inhibit the growth of VP_AHPND_ in vitro, and a similar mechanism would be proposed in vivo. However, the effect of secreted AMPs or bacterial components on the activation of the shrimp immune system, which contributes to retained survival, should be further elucidated.

Despite the apparent differences in environmental conditions between shrimp stomachs and mangrove forest soil, there were no clear patterns in the distributions of the tested AMP-related genes among *Bacillus* isolates from each origin. It is expected that the *Bacillus* present in the shrimp stomach is not specifically adapted to the shrimp stomach environment but rather that *Bacillus* that could be present in the external environment is ingested orally and colonizes the shrimp stomach. While some previous studies claimed that bacteria administered by feeding colonized shrimp [15,41], it is not clear whether supplied *Bacillus* in water can stably colonize the digestive tract of shrimp. A previous report showed very low colonization rates of *Bacillus* bacteria in the digestive tracts of shrimp, especially in earthen ponds [42]. For this reason, the field trial in this study was conducted with continuous administration of the test bacterium, but the optimization of dosing methods is needed.

In general, strains of VP_AHPND_ are halophilic, and the salinity in the water affects their virulence [43,44]. Experimental infections in this study also showed different mortality rates of shrimp in a salinity-dependent manner. However, in shrimp hatcheries, it may be difficult to reduce the salinity [45]. In contrast, inland water aquaculture of Pacific white shrimp uses low-salinity water for cultivation [46,47]. In this study, at each salinity, mortality during VP_AHPND_ challenge was lower in the experimental group using *B. subtilis* (K3). This indicates that the effects can be expected under a variety of environmental salinity conditions.

Given the definition of the term probiotic [5], it may not be appropriate to use the designation probiotic for the use of *Bacillus* in this study (exposure via immersion). However, shrimp are expected to take up bacteria in the water, as evidenced by the fact that oral infection of *Vibrio* is experimentally established using immersion [26]. Further research is needed on the dynamics of the supplied bacteria during immersion, but it might become possible to use the term probiotics for this strategy of the use of beneficial bacteria for shrimp in this method.

Although *Bacillus* bacteria have been isolated from the shrimp gut [14,15,48], previous analyses of the 16S rDNA-based microbiome show that the genus *Bacillus* is rarely the dominant genus in the shrimp gut [49], despite its strong inhibitory activity against other bacteria. In the environment of the shrimp digestive tract, it is likely that the balance of microbes is maintained among many bacterial species via bacterial competition or interrelationships with the host. Human studies and subsequent mouse model studies have shown that rare *Bacillus* in the gastrointestinal tract reduces the risk of infectious disease outbreaks [50]. Surely, the findings in mammals cannot be easily applied to shrimp, but there are phenomena that cannot be fully elucidated by sequencing microbiome analysis alone, and isolation of bacterial strains and subsequent in vitro and in vivo analysis remain useful.

This study was based on selected AMP-related genes for genetic analysis rather than whole genome analysis. We cannot deny that the isolates might harbor novel or overlooked AMP genes that contribute to bacterial inhibition. In addition, it remains unclear whether the differences in inhibitory activity between isolates in vitro are dependent on the amount of AMP secreted or the activity of each peptide molecule. Because of the limitations of this study, it is not certain that similar results can be obtained with other *Bacillus* isolates.

Importantly, selected *B. subtilis* (K3) isolate do not possess antibiotic resistance properties. It is suggested that the use of *B. subtilis* (K3) as a probiotic is safe for aquatic animal cultivation and suitable for producing fishery products for human consumption. In the future, the replacement of antibiotics with probiotics not only reduces the use of chemicals in agriculture, which leave residues in the environment, but also reduces bacteria harboring AMR, which reduces the transfer of antimicrobial-resistant bacteria to humans.

In conclusion, an isolate of *Bacillus* spp., which was obtained in this study, decreased the mortality of shrimp challenged with VP_AHPND_. The criterion to screen potential beneficial *Bacillus* spp. is useful for the search for probiotics for shrimp. This study also shows one of the possible mechanisms of the beneficial effects of probiotics on shrimp.

## Figures and Tables

**Table 1:** Primers used to detect the AMP-related genes: *bmyB* (bacillomycin L synthetase B), *fenD* (fengycin synthetase), *ituC* (iturin A synthetase C), *srfAA* (surfactin synthetase subunit 1), *bacA* (bacilysin biosynthesis protein) and *spaS* (subtilin).

Gene	AMP	Primer	Sequence (5′ → 3′)	Product Size (bp)
*fenD*	Fengycin	*fenD*_F	GGCCCGTTCTCTAAATCCAT	269
		*fenD*_R	GTCATGCTGACGAGAGCAAA	
*bmyB*	Bacillomycin	*bmyB*_F	GAATCCCGTTGTTCTCCAAA	370
		*bmyB*_R	GCGGGTATTGAATGCTTGTT	
*ituC*	Iturin	*ituC*_F	GGCTGCTGCAGATGCTTTAT	423
		*ituC*_R	TCGCAGATAATCGCAGTGAG	
*srfAA*	Surfactin	*srfAA*_F	TCGGGACAGGAAGACATCAT	201
		*srfAA*_R	CCACTCAAACGGATAATCCTGA	
*bacA*	Bacilysin	*bacA*_F	CAGCTCATGGGAATGCTTTT	498
		*bacA*_R	CTCGGTCCTGAAGGGACAAG	
*spaS*	Subtilin	*spaS*_F	GGTTTGTTGGATGGAGCTGT	375
		*spaS*_R	GCAAGGAGTCAGAGCAAGGT	

**Table 2:** Susceptibility of *Bacillus* isolates (K3) against antibiotics, including amoxicillin, oxytetracycline, sulfa-trimethoprim, doxycycline, erythromycin, gentamycin, enrofloxacin, tetracycline, ceftriaxone, streptomycin and norfloxacin.

Antibiotic	Disc Potency (µg)	*Bacillus* sp. Isolate K3	
		Zone Diameter (mm)	Interpretation
Amoxicillin	10	15.0	I
Ceftriaxone	30	38.5	S
Doxycycline	30	27.0	S
Gentamycin	10	16.5	S
Enrofloxacin	5	27.5	S
Erythromycin	15	21.0	S
Norfloxacin	10	31.0	S
Oxytetracycline	30	26.5	I
Streptomycin	10	14.0	I
Sulfa-trimethoprim	25	28.5	S
Tetracycline	30	28.0	S

**Table 3:** Presence of AMP genes in *Bacillus* isolates.

Isolate	Origin of Isolation	16S rRNA	*BacA*	*srfAA*	*ituC*	*fenD*	*spaS*	*bmyB*
K1	Shrimp	*B. methylotrophicus*	+	+	−	+	−	+
K2	Shrimp	*B. licheniformis*	−	+	−	−	−	−
K3	Shrimp	*B. subtilis*	+	+	−	−	−	−
K4	Shrimp	*B. subtilis*	+	+	−	−	−	−
K5	Shrimp	*B. licheniformis*	−	+	−	−	−	−
K6	Shrimp	*B. amyloliquefaciens*	+	+	+	+	−	+
K7	Shrimp	*B. amyloliquefaciens*	+	+	−	+	−	+
K8	Shrimp	*B. subtilis*	+	+	−	−	−	+
K9	Shrimp	*B. amyloliquefaciens*	+	+	−	+	−	+
K10	Shrimp	*B. subtilis*	+	+	−	+	−	+
K11	Shrimp	*B. vallismortis*	−	+	−	−	−	+
K12	Shrimp	*B. subtilis*	−	+	−	+	−	−
K13	Shrimp	*B. subtilis*	−	−	−	−	−	−
K14	Shrimp	*B. subtilis*	+	+	−	−	−	−
K15	Shrimp	*B. subtilis*	+	+	−	−	−	−
K16	Shrimp	*B. licheniformis*	−	+	−	−	−	−
K17	Shrimp	*B. subtilis*	+	+	−	+	−	+
K18	Shrimp	*B. subtilis*	+	+	−	−	+	−
K19	Shrimp	*B. subtilis*	+	+	−	−	−	+
K20	Shrimp	*B. cereus*	−	−	−	−	−	−
K21	Shrimp	*B. licheniformis*	−	+	−	−	−	−
K22	Shrimp	*B. flexus*	−	−	−	−	−	−
P1	Mangrove	*B. tequilensis*	+	+	−	−	−	−
P2	Mangrove	*B. amyloliquefaciens*	+	+	−	−	−	−
P3	Mangrove	*B. tequilensis*	+	+	−	−	−	−
P4	Mangrove	*B. amyloliquefaciens*	+	+	−	+	−	−
P5	Mangrove	*B. velezensis*	+	+	−	+	−	+
P6	Mangrove	*B. velezensis*	+	+	−	+	−	+
P7	Mangrove	*B. methylotrophicus*	−	−	−	−	−	−
P8	Mangrove	*B. velezensis*	−	−	−	−	−	−
P9	Mangrove	*B. firmus*	−	−	−	−	−	−
P10	Mangrove	*B. velezensis*	−	−	−	−	−	−

**Table 4:** Results of the field trial. Number of AHPND infection ponds, number of drain ponds, % average survival, total shrimp weight (kg), total feed (kg), and FCR of shrimp receiving *B. subtilis* (K3) and the control group.

Parameters	Control	*B. subtilis* (K3)
No. of ponds	30	30
No. of shrimp stocked/pond	600,000	600,000
No. of AHPND-positive ponds	10	2
Average survival rate (%)	68.1	94.6
Average FCR	1.67	1.45
Number of drain ponds	5	0
Final size (pcs/kg)	942.48	783.53
Total shrimp weight (kg)/pond	433.75	724.53
Total feed (kg)/pond	725.75	1053.90
No. of surviving shrimp/pond	408,801	567,604

## Data Availability

Data will be made available on request.

## Supplementary Materials

The following supporting information can be downloaded at: https://www.mdpi.com/article/10.3390/microorganisms11092176/s1.
