# Supplementary material for: Evaluation of Bacillus spp. as Potent Probiotics with Reduction in AHPND-Related Mortality and Facilitating Growth Performance of Pacific White Shrimp (Litopenaeus vannamei) Farms
Source: Microorganisms. 2023 Aug 29;11(9):2176. doi: 10.3390/microorganisms11092176 (PMC10537061; doi:10.3390/microorganisms11092176)

SUPPLEMENT DATA

Supplementary figures

Supplement data S1. Location of shrimp nursery farms in Vietnam.

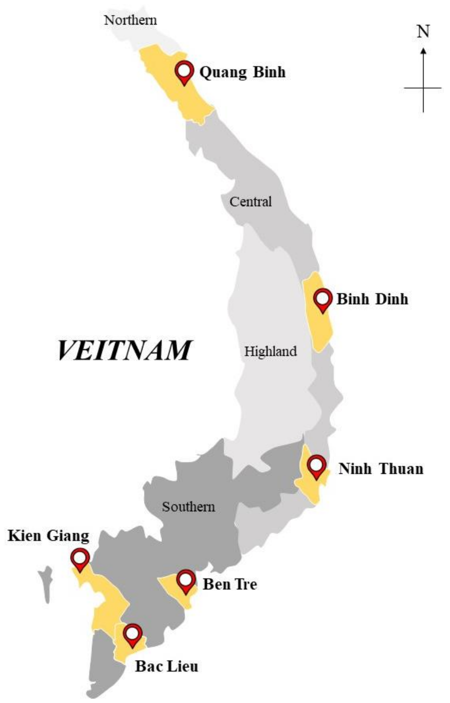

Supplement data S2. Survival of shrimp challenged by immersion with 10^4 CFU/mL of various AHPND-causing strains of *Vibrio parahaemolyticus* (strain RY, CT, TR, CP, SR, NK, and SK).

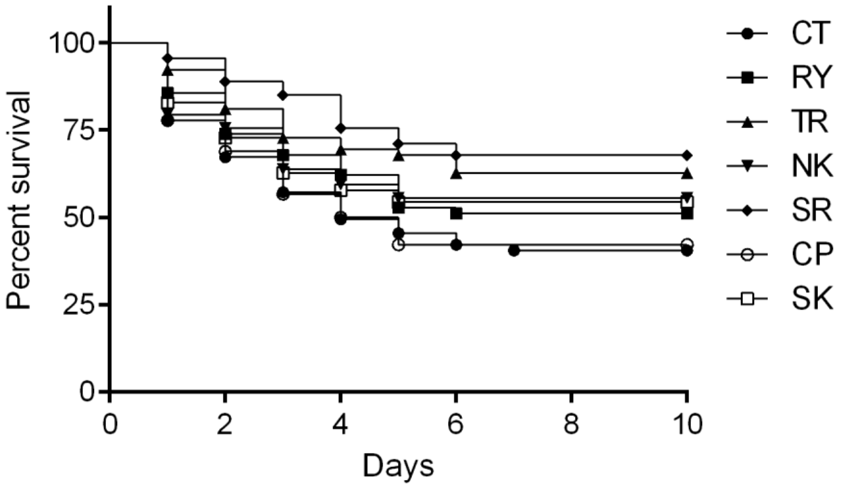

Supplement data S3.

PCR detection of VP$_{AHPND}$ of the hepatopancreas collected from moribund shrimp after challenged with different strains of VP$_{AHPND}$ (Tinwongger et al., 2014). VP$_{AHPND}$ collected from A) Chanthaburi (CT), B) Rayong (RY), C) Trat (TR), D) Nakhon Si Thammarat (NK), E) Surat Thani (SR), F) Chumphon (CP), and G) Songkhla (SK). 100 bp is the 100 bp DNA marker, -ve is the negative control.

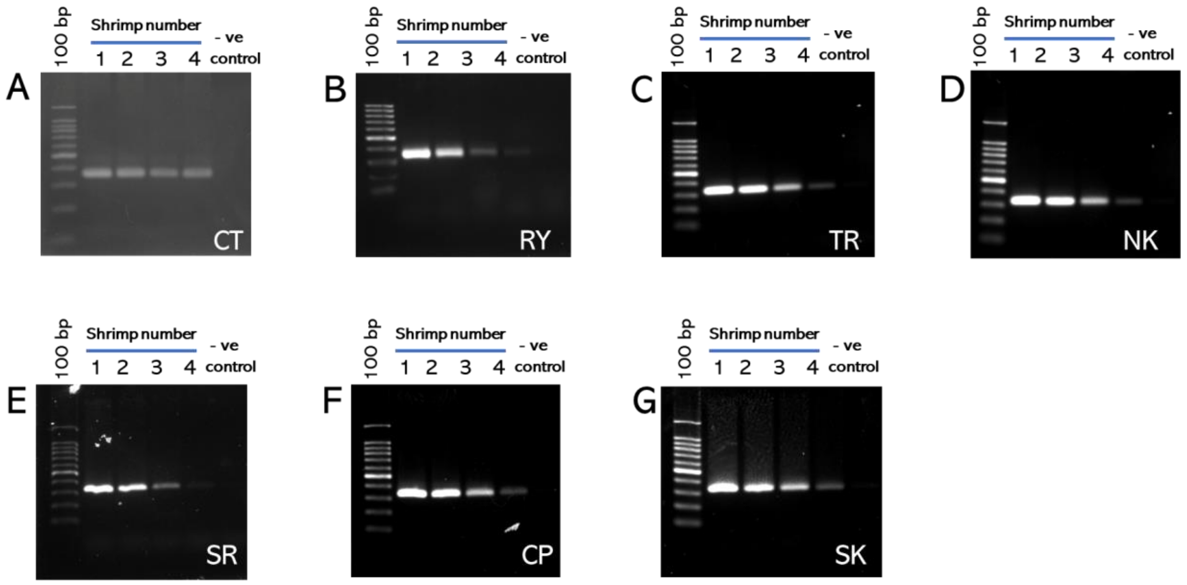

Supplement data S4.

Histomorphology determination (H&E staining) of the hepatopancreas collected from moribund shrimp after challenged with different strains of VP$_{AHPND}$ (Tinwongger et al., 2014). VP$_{AHPND}$ collected from A) Chanthaburi (CT), B) Rayong (RY), C) Trat (TR), D) Nakhon Si Thammarat (NK), E) Surat Thani (SR), F) Chumphon (CP), and G) Songkhla (SK).

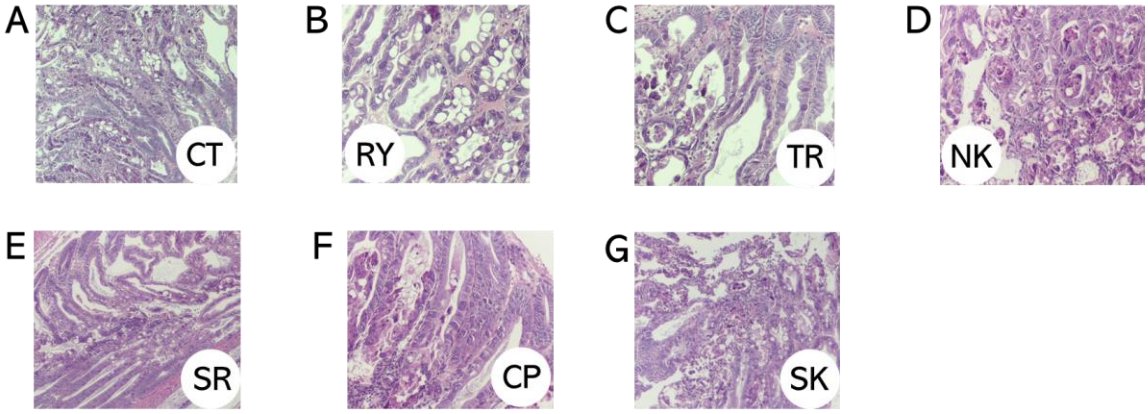

Supplement: Supplementary file 1 [file microorganisms-11-02176-s001.zip › microorganisms-2518665-supplementary.pdf]
